# Phagocytic and Bactericidal Properties of Channel Catfish Peritoneal Macrophages Exposed to *Edwardsiella ictaluri* Live Attenuated Vaccine and Wild-Type Strains

**DOI:** 10.3389/fmicb.2017.02638

**Published:** 2018-01-09

**Authors:** Adef O. Kordon, Hossam Abdelhamed, Hamada Ahmed, Joo Y. Park, Attila Karsi, Lesya M. Pinchuk

**Affiliations:** ^1^Department of Basic Sciences, College of Veterinary Medicine, Mississippi State University, Mississippi State, MS, United States; ^2^Department of Nutrition and Veterinary Clinical Nutrition, Faculty of Veterinary Medicine, Damanhour University, Damanhour, Egypt

**Keywords:** *Edwardsiella ictaluri*, live attenuated vaccines, catfish macrophages, phagocytosis, bacterial killing, complement

## Abstract

*Edwardsiella ictaluri* (*E. ictaluri*), a Gram-negative, intracellular, facultative bacterium, is the causative agent of enteric septicemia of catfish (ESC), which is one of the most significant diseases of farmed channel catfish. Macrophages have a critical role in major defense mechanisms against bacterial infections by migrating to the site of infection, engulfing and killing pathogens, and priming adaptive immune responses. Vaccination of catfish with *E. ictaluri* live attenuated vaccine (LAV) strains increased the efficiency of phagocytosis and bacterial killing in catfish peritoneal macrophages compared *in vitro* with macrophages from non-vaccinated fish. Recently, our group developed several protective LAV strains from *E. ictaluri*. However, their effects on the antigen uptake and bacterial killing in catfish macrophages have not been evaluated. In this study, we assessed the phagocytic and bactericidal activity of peritoneal macrophages in the uptake of *E. ictaluri* wild-type (WT) and two LAV strains. We found that phagocytosis of LAV strains was significantly higher compared to their WT counterpart in peritoneal macrophages. Moreover, the uptake of *E. ictaluri* opsonized with sera from vaccinated catfish was more efficient than when opsonized with sera from sham-vaccinated fish. Notably, catfish macrophages did not lose their phagocytic properties at 4°C, as described previously in mammalian and zebrafish models. Also, opsonization of *E. ictaluri* with inactivated sera from vaccinated and sham-vaccinated catfish decreased significantly phagocytic uptake of bacteria at 32°C, and virtually suppressed endocytosis at 4°C, suggesting the important role of complement-dependent mechanisms in catfish macrophage phagocytosis. In conclusion, our data on enhanced phagocytic capacity and effective killing ability in macrophages of vaccine strains suggested the LAVs’ advantage if processed and presented in the form of peptides to specific lymphocytes of an adaptive immune system and emphasize the importance of macrophage-mediated immunity against ESC. Furthermore, we showed the role of complement-dependent mechanisms in the phagocytic uptakes of *E. ictaluri* in catfish peritoneal macrophages at 4 and 32°C. Finally, LAV vaccine-induced bacterial phagocytosis and killing properties of peritoneal macrophages emphasized the importance of the innate immune responses in ESC.

## Introduction

*Edwardsiella ictaluri*, a Gram-negative, intracellular, facultative bacterium, is the causative agent of ESC, which is one of the most significant diseases of farmed channel catfish (Hawke et al., 1981; Miyazaki and Plumb, 1985; Wagner et al., 2006; Zhang and Arias, 2007). Klesius and Shoemaker (1999) developed a modified live *E. ictaluri* vaccine against ESC (commercialized later as Aquavac-ESC) that stimulated protective immunity delivered by bath immertion in juvenile catfish. Subsequent immersion studies showed effective protection in catfish fry, fingerlings, and eyed catfish eggs (Shoemaker et al., 1999, 2002, 2007; Wise et al., 2000), which also demonstrated that *E. ictaluri* LAVs induced cell-mediated immunity to protect catfish against ESC (Shoemaker and Klesius, 1997; Ellis, 1999) because *E. ictaluri* could survive and replicate in channel catfish macrophages (Booth et al., 2006). Furthermore, vaccination of catfish with LAVs resulted in the specific antibodies production that enhanced the bactericidal activity of macrophages (Shoemaker and Klesius, 1997).

Macrophages are professional phagocytes that have multiple functions in different species including immunity, inflammation, and tissue repair (Godwin et al., 2013). New evidence has accumulated on the progenitors of adult tissue resident macrophages, embryonic macrophages (Schulz et al., 2012; Hashimoto et al., 2013; Epelman et al., 2014; Hoeffel et al., 2015; Sheng et al., 2015). Professional phagocytes, including macrophages, in fish have a significant role in major defense mechanisms against bacterial infections as these cells can migrate to the site of infection and engulf and kill pathogens (Secombes and Fletcher, 1992; Esteban et al., 2015). Multiple studies documented strong phagocytic capability and bactericidal activity of anterior kidney macrophages against intracellular pathogens including parasites, yeast, and bacteria (Bennani et al., 1995; Dieter and Katharina, 1997; Esteban et al., 1998; Muñoz et al., 2000; Qiu et al., 2016). Importantly, macrophages are present normally in the peritoneal cavity of fish; therefore, the peritoneal macrophage approach has been documented well for *in vitro* immunologic studies in catfish (Jenkins and Klesius, 1998). Collection of peritoneal macrophages is relatively easy and does not require special isolation and purification procedures (Jenkins and Klesius, 1998). Moreover, phagocytes response to IP inflammation is easily observed and measured both qualitatively and quantitatively (Silva et al., 1989).

Peritoneal macrophages from sea bass had significantly greater phagocytic activity against bacteria, such as *Escherichia coli and Salmonella enterica serovar Typhimurium*, than did monocytes and macrophages from blood and anterior kidney, respectively (Esteban and Meseguer, 1997). Furthermore, a higher number of phagocytosed bacteria was observed in macrophages than other phagocytic cells in the peritoneal cavity of sea bass (Do Vale et al., 2002). Phagocytosis of *Yersinia ruckeri* in peritoneal macrophages was significantly greater compared to the phagocytic activity of neutrophils in rainbow trout (*Oncorhynchus mykiss*) (António et al., 1998). Winter flounder (*Pleuronectes americanus*) peritoneal macrophages were capable of engulfing formalin-fixed bacteria (*Y. ruckeri* and *Bacillus cereus*) after a short exposure (Bodammer and Robohm, 1996). The phagocytic and microbicidal activity of peritoneal macrophages were described in rohu (*Labeo rohita*) and walking catfish (*Clarias batrachus*) (Mamnur Rashid et al., 2002; Awasthi et al., 2015). Shoemaker et al. (1997) evaluated the role of peritoneal macrophages in immunity to ESC after infection with live *E. ictaluri*. Phagocytic and bactericidal activity was significantly greater in macrophages from fish immunized with AL-93-75 compared to their counterparts from pathogen-free susceptible fingerling catfish (Russo et al., 2009). Interestingly, opsonization of *E. ictaluri* with immune serum significantly enhanced the killing ability of macrophages from susceptible fish (Shoemaker et al., 1997; Russo et al., 2009).

One of the major manifestations of immunological autophagy is the destruction and elimination of invading pathogens (Deretic et al., 2013; Mizumura et al., 2014). Recently, autophagy has been described in fish (van der Vaart et al., 2012; Yabu et al., 2012; García-Valtanen et al., 2014). Engulfment of microbial prey as part of autophagy is initiated at the plasma membrane of the macrophage where a vast repertoire of phagocytic receptors, in particular, CRs, recognize the bacterial surface directly or indirectly through deposition of serum opsonins such as IgG or the complement protein C3b (Herre et al., 2004; Patel and Harrison, 2008). Complement activation is a tightly regulated process that may proceed through three distinct pathways: the alternative pathway, classical pathway, and the lectin-dependent pathway. However, each pathway converges on the complement protein C3b to generate the bioactive components C3a and C3b (Merle et al., 2015). Similar to higher vertebrates, the complement system in teleost fish can be activated through all three pathways and shows many effector functions identified in mammalian complement system, such as opsonization, anaphylatoxic leukocyte stimulation, and target cell killing (Holland and Lambris, 2002; Nakao et al., 2011). However, some complement components in teleost fish are present in multiple active isoforms, in particular the key complement component, C3, is present in several isoforms produced by different genes (Sunyer et al., 1996, 1997; Nakao et al., 2000; Boshra et al., 2006). In addition, catalytic residues of proteins of complement are different in teleost fish (Nakao and Yano, 1998). Furthermore, unlike in mammals, fish complement components are active at low temperatures and show higher magnitude providing broader recognition of foreign substances in fish (Sunyer and Tort, 1995; Sunyer and Lambris, 1998; Boshra et al., 2006). It is well documented that opsonization of bacteria with serum proteins and fixation of complement are formidable barriers that must be overcome to establish infection (Flannagan et al., 2015). Whereas the specific signaling molecules can vary, the requirement for actin remodeling in the engulfment process is absolute (Greenberg et al., 1991; Tse et al., 2003). The lumen of phagolysosome is an inhospitable environment for intracellular pathogens (Flannagan et al., 2015). Lysosomal proteases that display a range of functions that shape the cellular immune response in macrophages, antimicrobial effectors that promote killing of phagocytosed pathogens through direct proteolytic attacks (Muller et al., 2014), and other active substances comprise part of the macrophages antimicrobial arsenal that exerts microbicidal effects by compromising directly bacterial membranes or promoting the production of immunomodulatory compounds (Weinrauch et al., 1996; Wu et al., 2010).

Recently, our research group has determined that *evpB* gene in the Type VI secretion system (T6SS) operon is differentially regulated during *in vitro* iron-restricted conditions (Dumpala et al., 2015). We constructed *Ei*Δ*evpB* strain by in-frame deletion of *evpB* gene and found that *E. ictaluri* is completely attenuated in catfish fingerlings and fry. Vaccination with *Ei*Δ*evpB* did not cause mortality in fingerlings (100% survival) and low 3–4% mortality in fry catfish after WT *E. ictaluri* challenge (Nho et al., 2017). Our finding corroborated an earlier study showing that *evpB* plays a key role in *E. tarda* pathogenesis (Zheng and Leung, 2007). Furthermore, our laboratory reported that genes encoded tricarboxylic acid cycle (*sdhCfrdA*) and one-carbon metabolism (*gcvP*) were essential for *E. ictaluri* virulence (Dahal et al., 2013). Similarly, we introduced an in-frame deletion of glycine dehydrogenase (gcvP), succinate dehydrogenase (sdhC), and fumarate reductase (frdA) genes in *E. ictaluri* 93–146 strain and named it as ESC-NDKL1 (EiΔgcvPΔsdhCΔfrdA) (Nho et al., 2017). Vaccination of catfish fingerlings with ESC-NDKL1 showed similar 100% survival rates as *Ei*Δ*evpB*, however, challenge of fry with ESC-NDKL1 showed moderatly elevated 3–4% mortality rates (Nho et al., 2017). The purpose of our study was to compare the phagocytic and bacterial killing activity of channel catfish peritoneal macrophages against *E. ictaluri* WT and two LAV strains in the presence of sera obtained from vaccinated fish. Increased phagocytic capacity and killing ability of macrophages against opsonized LAV strains will support the importance of macrophage-mediated immunity against ESC in catfish.

## Materials and Methods

### Animals

The fish hatchery at the College of Veterinary Medicine, Mississippi State University, provided SPF channel catfish, which were maintained at 25–28°C. All fish experiments were performed based on a protocol approved by the Mississippi State University Institutional Animal Care and Use Committee (IACUC). Tricaine methanesulfonate (MS-222, Western, Chemical, Inc.) was used to sedate (100 mg/ml) and euthanize (400 mg/ml) the catfish. Samples were obtained as described below.

### Cell Harvesting

Channel catfish (250–300 g) were used in this study. Peritoneal macrophages were harvested as described previously as a simple reliable method to obtain tissue resident macrophages that displayed high oxygen production and phagocytic ability (Jenkins and Klesius, 1998). Briefly, 1 ml squalene (Sigma–Aldrich, St. Louis, MO, United States) was injected IP in sedated catfish. After 4 days of injection, catfish were sedated, the peritoneal area wiped with 70% ethanol, and cold, sterile phosphate-buffered saline (PBS) injected IP via a three-way valve attached to a syringe, 18-gauge needle, and Tygon tubing. The valve was then closed, the fish’s abdominal region was massaged gently, and a cell suspension collected into a centrifuge tube placed on ice. Additional cold PBS was injected into the peritoneal cavity until IP fluid was clear.

### Bacterial Strains and Opsonization

Bacterial strains used in this work are listed in **Table 1**. *E. ictaluri* 93–146 WT strain and vaccine strains were cultured in BHI agar or broth (Difco, Sparks, MD, United States) and incubated throughout the study at 30°C. Each vaccine strain was labeled with bioluminescence by transferring pAK*gfplux*1 from an *E. coli* donor strain (SM10aaa*pir*) by conjugation as described previously (Karsi and Lawrence, 2007). When required, media were supplemented with following antibiotics and reagents; ampicillin (Amp: 100 mg/ml), and colistin sulfate (Col: 12.5 mg/ml, Sigma–Aldrich, St. Louis, MN, United States).

**Table 1:** Bacterial strains and plasmids.

Bacterial strain	Description	Reference
*Edwardsiella ictaluri* Strain 93–146	WT; pEI1^+^; pEI2^+^; Col^r^; pAK*gfplux*1	Lawrence et al., 1997
ESC-*Ei*Δ*evpB*	93–146 derivative; Δ*evpB*; pAK*gfplux*1	Dr. Attila Karsi Mississippi State University
ESC-NDKL1	93–146 derivative; Δ*gc*vPΔ*sdhC*Δ*mdh*; pAK*gfplux*1	Dr. Mark Lawrence Mississippi State University

Overnight cultures of *E. ictaluri* and vaccine strains were grown and prepared as described above, followed by two PBS washes before opsonization with channel catfish serum for 30 min at room temperature. Opsonized bacteria were then used for antigen uptake and infection experiments.

### Fish Vaccination and Serum Collection

A total of 100 SPF channel catfish fingerlings (6-month-old) with fully developed innate and adaptive immune systems (Patrie-Hanson and Ainsworth, 1999; Petrie-Hanson and Ainsworth, 2001; Rombout et al., 2005; Zapata et al., 2006) were stocked into four 40-L tanks (25 fish per tank) with a continuous water flow and aeration. Tanks were assigned randomly to *Ei*Δ*evpB*, ESC-NDKL1, *E. ictaluri* WT (positive control), and sham-vaccinated (negative control) groups. The fish were fed twice daily and acclimatized for 1 week. The water temperature was maintained at 24–26°C throughout the trial. After a week of acclimation, fish were exposed *Ei*Δ*evpB*, ESC-NDKL1, and WT *E. ictaluri* 93–146 by immersion challenge as described previously (Abdelhamed et al., 2013). Briefly, 100 ml of overnight cultures were added to 10 L water to yield infection dose of approximately 3.67 × 107 CFU/ml of water. The negative control group was immersion challenged with BHI broth.

Blood samples were collected from caudal vein of 10 fish at 14 and 21 days post-infection and were allowed to coagulate overnight at 4°C. Serum was obtained by centrifugation at 8000 rpm for 10 min.

### Phagocytosis and Flow Cytometry

Harvested cells were washed three times in PBS at 2000 rpm for 10 min at 24°C and resuspended in CCMM, which included RPMI [(RPMI 1640 sans phenol red and L-glutamine, (0.407 mM magnesium sulfate and 0.424 mM calcium nitrate) Lonza, Walkersville, MD, United States) containing 1× glutamine substitute (GlutaMAX-I CTS, Gibco, Invitrogen Corporation, Carlsbad, CA, United States)], 15 mM HEPES buffer (GIBCO), in 0.18% sodium bicarbonate solution (GIBCO), 0.05 mM 2-beta-mercaptoethanol (all from Sigma Chemical Co., St. Louis, MO, United States), and 5% HI pooled channel catfish serum. Cells were counted using a hemocytometer and trypan blue exclusion. Cell suspension was transferred into a 6-well plate (Fisher Scientific, Pittsburgh, PA, United States), and the phagocytic capacities of peritoneal macrophages were determined by addition of GFP transformed bacterial strains in 1: 50 ratio and incubated at 32 (active uptake) and 4°C (background levels of endocytosis, negative control) for 2 h in the dark. Following incubation, cells were harvested with cell scrapers (Fisher Scientific, Pittsburgh, PA, United States), and washed three times by centrifugation in cold PBS and analyzed using FACSCalibur (Becton Dickinson), as follows. After setting a gate on large granular cells, the LAVs incorporation was measured and analyzed by using FlowJo 7.6.4 Software (Tree Star Inc.). To inhibit actin formation selectively, catfish macrophages were incubated for 10 min in the presence of CCD (2.5 μg/ml, Sigma–Aldrich, St. Louis, MO, United States) before the addition of LAVs bacterial strains (Watts and Marsh, 1992). To determine differences between treatments, MFI of engulfed bacteria in catfish peritoneal macrophages was analyzed using single histogram and overlay histogram statistics.

### Cytospin and Light Microscopy

Peritoneal macrophages were incubated in the dark with GFP-labeled WT *E. ictaluri* and LAVs at 32 and 4°C for 2 h. Cells were then harvested, washed, and the cytospin preparations applied at 500 rpm for 1 min with a Cyto-Tek centrifuge machine. All samples were fixed and stained with Wright’s stain (Hemacolor, Merck) as described (Do Vale et al., 2002), analyzed, and then photographed with Olympus BX60 microscope (Olympus U-TV1 X) and Infinity software.

### Bacterial Killing Assay

The bacterial killing assay was performed as described previously with some modifications (Booth et al., 2006; Russo et al., 2009). Briefly, harvested peritoneal macrophages were washed with PBS by centrifugation, resuspended in CCMM, and transferred to 96-well plates. WT strain and LAVs were added to catfish macrophages in 1:1 ratio followed by centrifugation at 1500 rpm for 5 min at 24°C to compact cells and bacteria and then incubated at 32°C for 1 h. After incubation, plates were centrifuged at 2000 rpm for 7–10 min, and supernatants removed. Next, cell pellets were resuspended in CCMM containing 100 μg/ml gentamicin (Gibco, Life Technologies, Grand Island, NY, United States) to kill extracellular bacteria, incubated at 32°C for 1 h, and washed by centrifugation in PBS. After washing, plates were incubated at 32°C for 10 and 24 h in CCMM containing 10 μg/ml gentamycin. For each time point, colony-counting method was performed, as follows. Macrophages were lysed with 1× Triton X-100 (Sigma, St. Louis, MO, United States), as described (Russo et al., 2009). Lysed macrophages were diluted in PBS and plated on a selective medium, and incubated at 32°C for 48 h.

### Statistical Analysis

The significance of the differences between means was established by one-way ANOVA and two-way ANOVA procedures with Tukey’s test in SAS for Windows 9.4 (SAS Institute Inc., Cary, NC, United States) to evaluate differences in MFIs. The level of significance for all tests was set at *P* < 0.05.

## Results

### Active Phagocytic Uptake of *E. ictaluri* LAVs in Peritoneal Macrophages

**FIGURE 1:**
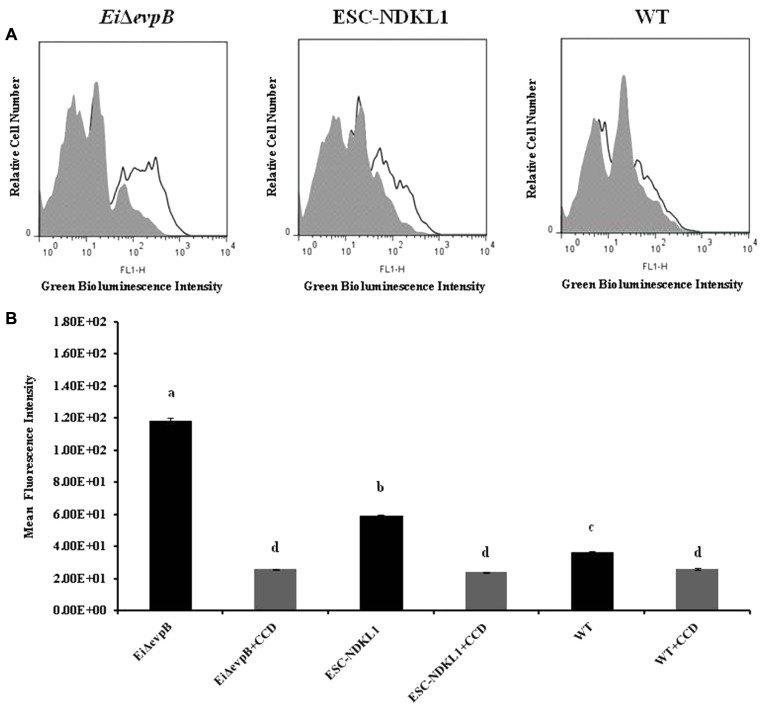
Active phagocytic uptake of *Ei*Δ*evpB* and ESC-NDKL1 strains in catfish peritoneal macrophages. **(A)** Original flow data by using overlay histogram statistics: Black line histograms indicate uptake of the LAVs and WT strains at 32°C. Gray histograms indicate antigen uptake in the presence of CCD at 32°C. **(B)** Statistical analysis of the phagocytic uptake mean of fluorescent intensity (MFI). Black filled columns in the graph show MFI of LAVs and WT strains uptake at 32°C. Gray filled columns show MFI of antigen uptake in the presence of CCD at 32°C. The data represent the mean of MFI of macrophage phagocytic uptake from five fish ± SD. The letters (a,b,c,d) show the significant differences between treatments (*P* < 0.05).

In this study, we evaluated endocytic uptake of *E. ictaluri* and the LAV strains in catfish peritoneal macrophages (**Figure 1**). To ensure active phagocytosis in macrophages, we measured the uptake of the GFP-labeled bacteria at 32°C and background endocytosis levels at 4°C. Also, we assessed the intensity of phagocytosis in peritoneal macrophages pre-incubated with the phagocytosis inhibitor, CCD (**Figures 1A,B**). The phagocytic intensity levels of both LAV strains at 32°C were significantly higher compared to the endocytosis of WT *E. ictaluri* in catfish peritoneal macrophages (**Figure 1B**). However, the uptake of *Ei*Δ*evpB* showed a significant increase compared to its ESC-NDKL counterpart (**Figure 1B**). Significant decreases but not the complete inhibition of uptake of LAVs and WT *E. ictaluri* were evident in the presence of actin formation inhibitor CCD (**Figure 1B**) and at 4°C (data not shown). In conclusion, active uptake of LAV strains was significantly higher compared to their WT counterpart in peritoneal macrophages at both temperatures.

**FIGURE 2:**
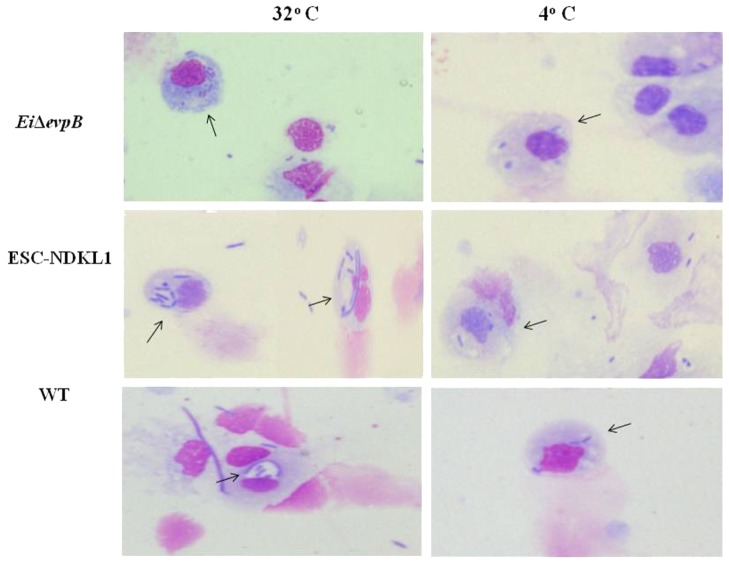
Active uptake of ESC vaccine strains in catfish peritoneal macrophages shown by light microscopy. The column on the left shows phagocytosis of *E. ictaluri* strains at 32°C, and the right column shows phagocytosis *E. ictaluri* strains at 4°C. Arrows indicate intracellular, engulfed bacterial cells in the cytoplasm and phagosomes of peritoneal macrophages.

The engulfed intracellular WT and the LAV strains were evident in catfish peritoneal macrophages at both 32 and 4°C temperatures assessed, confirming our previous observation that catfish peritoneal macrophages did not lose their phagocytic properties at 4°C (**Figure 2**). In summary, our data showed the significant increases in bacterial uptake at 32°C compared to 4°C, the inhibitory effects of CCD, and the presence of engulfed intracellular bacteria demonstrated an active uptake of WT and LAVs in catfish peritoneal macrophages. Furthermore, both LAVs were taken more vigorously by macrophages compared to their WT counterpart, suggesting the LAVs advantage for elimination and/or processing for antigen presentation.

### Active Uptake of *E. ictaluri* Opsonized with the LAVs-Induced Immune Sera in Peritoneal Macrophages

**FIGURE 3:**
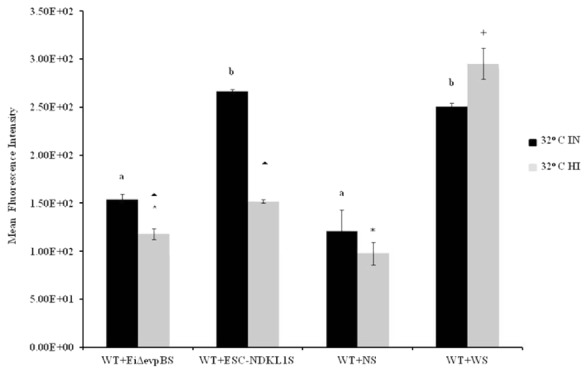
Active uptake of *E. ictaluri* LAVs opsonized with IN and HI sera from challenged fingerlings in catfish peritoneal macrophages at 32°C. *Ei*Δ*evpB* and ESC-NDKL1S indicate serum of vaccinated fish with *Ei*Δ*evpB* and ESC-NDKL1, respectively; WS indicates serum from fish challenged with WT, and NS indicates serum from control fish. ^a,b^Presence of letters on top of bars indicates group differences in uptakes with bars with different letters being different from each other and from bars without a letter designation (*P* < 0.05). ^∗^Indicates treatment differences in the uptakes (*P* < 0.05). ^∧+^Indicate group differences in the uptakes (*P* < 0.05). The data represent the mean of MFI of macrophage phagocytic uptake from five fish ± SD.

**FIGURE 4:**
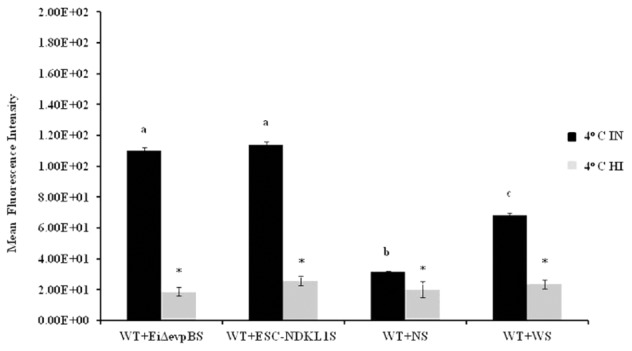
Active uptake of *E. ictaluri* LAVs opsonized with IN and HI sera from challenged fingerlings in catfish peritoneal macrophages at 4°C. ^a,b,c^Presence of letters on top of bars indicates treatment differences in the uptakes with bars with different letters being different from each other and from bars without a letter designation (*P* < 0.05). ^∗^Indicates treatment differences in the uptakes (*P* < 0.05). The data represent the mean of MFI of macrophage phagocytic uptake from five fish ± SD.

Opsonization of WT *E. ictaluri* with sera from fingerlings vaccinated with both LAVs and non-vaccinated control was applied to determine the protective effect of antibodies and complement on *E. ictaluri* phagocytosis in peritoneal macrophages. The MFI of phagocytosis of WT *E. ictaluri* opsonized with intact (IN) and HI sera from vaccinated and control catfish was assessed by flow cytometry (**Figure 3**). We found significant group- and treatment-related differences in the intensity of phagocytic uptake of WT *E. ictaluri* compared to LAV’s induced phagocytosis in catfish peritoneal macrophages. Namely, peritoneal macrophages had increased significantly higher uptake of opsonized bacteria compared to the non-opsonized *E. ictaluri* (data not shown). Phagocytosis of WT *E. ictaluri* opsonized with ESC-NDKL1-derived serum was increased significantly compared to the *Ei*Δ*evpB*, and IN serum-opsonized bacteria and did not differ from the uptake of WT *E. ictaluri* opsonized the WT-derived serum (**Figure 3**). Additionally, we found significant increases in phagocytosis of opsonized bacteria at 4°C showing similar patterns to the increases at 32°C (**Figure 4**). These patterns of uptake at 4°C, however, had significantly less phagocytic activity compared to phagocytosis occurring at 32°C (**Figure 3**). As expected, our results indicated that *E. ictaluri* opsonized with sera from vaccinated fish were taken by peritoneal macrophages more efficiently compared to the bacterium opsonized with sera from control animals, suggesting an important role of humoral responses at the early step of *E. ictaluri* antigen presentation.

### Active Uptake of *E. ictaluri* Opsonized with the LAVs-Induced Heat-Inactivated Immune Sera

Opsonization of WT *E. ictaluri* with HI sera from fingerlings vaccinated with both LAVs and non-vaccinated controls was utilized to determine the effect of complement on *E. ictaluri* phagocytosis in peritoneal macrophages. *E. ictaluri* opsonized with complement-inactivated sera derived from normal and ESC-NDKL-challenged fingerlings was phagocytosed at significantly lower rates compared to the bacteria treated with *Ei*Δ*evpB* and WT treatment derived sera (**Figure 3**). There were no significant differences in the intensity of phagocytic uptake of bacteria treated with HI *Ei*Δ*evpB* and WT sera (**Figure 3**). Significant increases in the levels of phagocytosis were evident in all *E. ictaluri* treatment groups compared to the uptake of untreated bacterial cells (data not shown). However, inactivation of complement did not affect the uptake of WT serum opsonized *E. ictaluri* in peritoneal macrophages (**Figure 3**). Our results showed clearly that active uptakes of *E. ictaluri* and LAVs candidates in catfish peritoneal macrophages at 32°C were mediated through the complement-dependent pathway.

We assessed phagocytic activity in catfish peritoneal macrophages exposed to WT *E. ictaluri* treated with IN and HI sera obtained from the challenged and control fish at 4°C (**Figure 4**). Phagocytic uptake of bacteria opsonized with sera obtained from fish challenged with both LAVs did not show significant differences. However, they did express significant increases compared to *E. ictaluri* opsonized in the presence of HI sera from challenged and control fish (**Figure 4**). As expected, phagocytosis of *E. ictaluri* opsonized with sera from control animals was significantly lower compared to the uptake of bacteria opsonized with sera from all challenged fish groups (**Figure 4**). Notably, phagocytosis in peritoneal macrophages exposed to *E. ictaluri* opsonized with HI sera obtained from the challenged and control fish showed dramatically decreased background levels of phagocytic activity (**Figure 4**). These results indicate strongly that complement was a crucial role in phagocytosis of *E. ictaluri* and LAVs candidates by peritoneal macrophages.

### Macrophages Killing of *E. ictaluri* Opsonized with the LAVs-Induced Immune Sera

**FIGURE 5:**
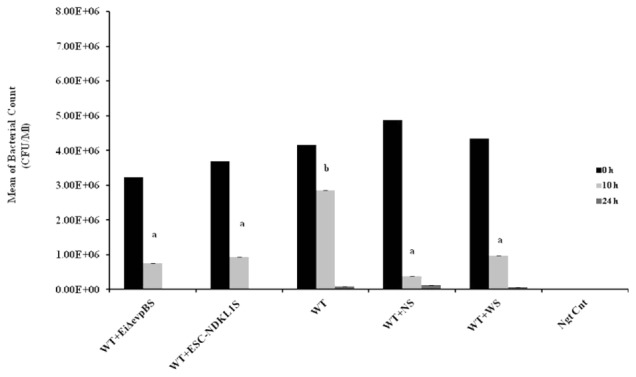
Bacterial killing of *E. ictaluri* LAVs opsonized with sera from challenged fingerlings in catfish peritoneal macrophages. Black columns indicate the colony numbers at 0 h, and gray columns indicate the colony numbers at 10 h, dark gray color columns indicate the colony numbers after 24 h of *in vitro* infection. ^a,b^Presence of letters on top of bars indicates group differences in the uptakes with bars with different letters being different from each other and from bars without a letter designation (*P* < 0.05). The data represent the mean of MFI of macrophage phagocytic uptake from five fish ± SD.

To examine how effective peritoneal macrophages are at destroying ingested bacteria, we performed the bacterial killing assay with *E. ictaluri* opsonized with IN sera from fish challenged with *E. ictaluri* LAVs, the WT strain, and control non-vaccinated fish (**Figure 5**). Initial numbers of colonies in all groups exposed to *E. ictaluri* did not show significant differences. However, the bacteria-killing capacity of peritoneal macrophages exposed to non-opsonized WT *E. ictaluri* was significantly lower compared to their counterparts treated with opsonized bacteria (**Figure 5**). No significant differences in killing capacity were evident in macrophages exposed to *E. ictaluri* treated with control, WT, or LAV-challenged fish derived sera following 10 h exposure (**Figure 5**). Numbers of bacterial colonies did not differ significantly in all experimental groups exposed to opsonized *E. ictaluri* 24 h after infection. However, consistent numerical increases in numbers of colonies were evident in peritoneal macrophages exposed to the WT strain, *E. ictaluri* opsonized with sera derived from control, and the WT-infected fish compared to their counterparts treated with bacteria opsonized with both LAVs-induced immune sera (**Figure 5**). The virtually absent bacterial colonies in peritoneal macrophages exposed to the sera from fish challenged with *E. ictaluri* LAVs indicated that peritoneal macrophages efficiently killed LAVs strains after 24 h of *in vitro* infection. No bacterial colonies were evident in the negative control of uninfected macrophages (**Figure 5**). The use of HI sera in the bacterial killing assay, in general, showed the patterns that did not differ significantly from IN serum–treated bacteria (data not shown).

## Discussion

Recent studies show that monocytes and macrophages are potent APCs that prime naïve T cells, and initiate adaptive cellular and humoral immune responses (Sakaguchi et al., 1995, 1996; Asano et al., 1996). Phagocytosis, which depends on membrane–cytoskeleton interactions, is an important step that mediates innate immune recognition by professional antigen presenting cells (APCs) and triggers adaptive immune responses (Sakaguchi et al., 1995). The current research aimed to assess the phagocytic and bactericidal activity of peritoneal macrophages in the uptake of WT *E. ictaluri* and two LAV candidate strains. Our conclusions agree with those of multiple studies that document strong phagocytic capability and bactericidal activity of peritoneal macrophages against intracellular pathogens (Esteban and Meseguer, 1997; António et al., 1998; Do Vale et al., 2002; Awasthi et al., 2015). We documented enhanced vaccine strains phagocytic capacity and effective bacterial killing ability of catfish peritoneal macrophages *in vitro*. Interestingly, the intensity of *Ei*Δ*evpB* LAV pahgocytic uptake was significantly higher compared to the ESC-NDKL1 LAV uptake in catfish peritoneal macrophages suggesting its advantage to be destroyed and processed into peptides by the APCs compared to ESC-NDKL1 LAV strain. Our previous report showed that catfish fry vaccinated with ESC-NDKL1 had higher mortality rates compared to the fry vaccinated with in *Ei*Δ*evp* LAV in E. *ictaluri* challenge (Nho et al., 2017). However, more research should be done to establish the necessary mechanistic framework for the LAV-dependent pathogenesis in catfish fry and fingerlings. Several earlier reports in humans and other mammals showed high-intensity antigen uptake at 37°C and low background levels of endocytosis at 4°C in professional APCs (Boyd et al., 2004; Ammari et al., 2014). Hohn et al. (2009) confirmed the data obtained in the mammalian studies on the low background levels of *E. ictaluri* phagocytosis at 4°C by using zebrafish anterior kidney/monocyte/macrophage/granulocyte phagocytes. However, contrary to the data derived from mammals, optimal conditions of antigen uptake in fish professional APCs are not well described yet. In contrast to previous observations, we report that active bacterial uptake in catfish peritoneal macrophages was detected at 32 and 4°C with significantly higher intensity at 32°C. Differences from earlier observations in our study regarding the intensity of active phagocytosis at 4°C could be due to several factors. First, there are phenotypic and functional differences between species in particular macrophages, and between APCs. Second, there are some fish species-specific differences in APC functions. Finally, monocytes, macrophages, and other professional phagocytes differ in the antigen uptake capacity due to their different locations and functions.

Importantly, our data agree with and contribute to the previous report that phagocytosis of LAV strains and WT *E. ictaluri* at 32°C was inhibited significantly in the presence of the actin formation inhibitor CCD, suggesting active uptake of *E. ictaluri* strains in catfish peritoneal macrophages (Hohn et al., 2009; Ammari et al., 2014). In particular, similar results were obtained with the uptake of WT *E. ictaluri* in the presence of CCD by zebrafish kidney phagocytes in the presence of CCD (Hohn et al., 2009). Also, a recent study showed that endocytosis of FITC-OVA in bovine monocytes was decreased significantly in the presence of CCD (Tomoda et al., 1989). In addition to the significantly increased intensity of bacterial phagocytosis at 32°C and substantial inhibitory effect of CCD on the endocytic uptake, we demonstrated the presence of engulfed intracellular bacteria in the cytoplasm and phagosomes of peritoneal macrophages, thus confirming the active endocytic mechanisms of WT *E. ictaluri* and LAVs in catfish APCs. Furthermore, both LAVs were endocytosed more vigorously by peritoneal macrophages compared to their WT counterpart suggesting the LAVs advantage to be processed and presented in the form of peptides to specific lymphocytes, and subsequently destroyed.

Phagocytosis is a receptor-mediated process, and these receptors are classified into two groups: non-opsonic receptors (e.g., Dectin-1 and CD36) and opsonic receptors (e.g., FcγRIIA and Mac-1) (Araki et al., 1996; Schlam et al., 2015; Levin et al., 2016). Non-opsonic receptors can recognize directly and bind to chemical structures present on the surface of pathogens, whereas opsonic receptors can recognize indirectly phagocytic targets via binding to immunoglobulins (e.g., IgG) or complement C3b (Flannagan et al., 2012; Levin et al., 2016). Opsonin C3b molecules are generated by complement activation, bind covalently to the pathogen surface to create a destruction by phagocytes, which have receptors [e.g., CR1 (CD35) and CRIg] for complement C3b protein (Erdei et al., 2016). In this study, we assessed the role of complement and antibodies in active uptake of *E. ictaluri* by peritoneal macrophages in catfish at 4 and 32°C. As expected, our results indicated that *E. ictaluri* opsonized with sera from vaccinated fish was endocytosed by peritoneal macrophages more efficiently compared to the bacteria opsonized with sera from control animals, suggesting an important role of secondary humoral responses at an early stage of LAVs antigen presentation. Our results agree with previous reports on the enhanced phagocytosis of bacteria in the presence of immune sera from vaccinated fish. Namely, Russo et al. (2009) showed that opsonization of *E. ictaluri* with serum from vaccinated fish augmented the *in vitro* phagocytic ability of macrophages in catfish. Also, another study (Esteban and Meseguer, 1997) reported that macrophages from sea bass showed greater phagocytic activity against opsonized bacteria. Recently, human monocyte-derived macrophages infected with *Francisella tularensis* showed 40 times more phagocytic activity in the presence of serum (Dai et al., 2013).

To confirm the significant contribution of complement-dependent mechanisms in the phagocytosis of *E. ictaluri*, we examined the uptake of the bacteria opsonized with HI sera from fish vaccinated with ESC-LAVs. We reported significant decreases in phagocytosis activity of peritoneal macrophages at both temperatures, which suggest the importance of CR ligation in *E. ictaluri* phagocytosis. Notably, the intensity of bacterial endocytosis was reduced dramatically to virtually background levels at 4°C, suggesting that bacterial uptake in catfish peritoneal macrophages at low temperatures, unlike that at 32°C, is predominantly CR-mediated. In contrast, the increased phagocytic activity of bacteria opsonized with HI WT serum in macrophages was evident at 32°C, suggesting a dominant role of the opsonic Fc receptors. Our findings are in agreement with earlier reports on the role of complement opsonic receptors in the endocytic activity of professional phagocytes. Namely, phagocytic activity of rainbow trout macrophages decreased significantly when *Mycobacterium marine* was treated with HI serum (Shih-Chu et al., 1998). HT serum also suppressed significantly phagocytosis of *Staphylococcus aureus* and zymosan particles in human macrophages (Peterson et al., 1977; Shih-Chu et al., 1998; Mankovich et al., 2013). Heat inactivation disturbs the complement cascade and removes C3 opsonins thereby decreasing phagocytic activity in monocyte-derived human macrophages (Chan et al., 2001). Taken together, various data indicate the importance of complement molecules engagement for efficient phagocytosis of bacterial cells. Our results also demonstrate that complement-mediated phagocytosis is temperature dependent in catfish peritoneal macrophages. Our data are in agreement with several previous observations that complement system in teleost fish is functionally active at low temperatures suggesting the enhanced complement-dependent phagocytosis compared to the mammalian counterpart (Sunyer and Tort, 1995; Sunyer and Lambris, 1998; Boshra et al., 2006).

Finally, we applied a bacterial killing assay to assess the efficacy of the opsonized *E. ictaluri* destruction compared to the killing ability of the non-opsonized WT bacterial strain. We confirmed the previously reported observation by Shoemaker et al. (1997) on the effective killing of opsonized *E. ictaluri* by catfish peritoneal macrophages compared to the phagocytes exposed to the non-opsonized WT strain. Our bacterial killing data in peritoneal macrophages showed that the numbers of bacterial colonies in peritoneal macrophages exposed to the opsonized WT *E. ictaluri* were significantly reduced compared to the phagocytes exposed to the non-opsonized WT strain showing significant differences at time 10 h post-exposure. In our study, the intensity of the immune sera opsonized WT *E. ictaluri* uptakes did not correlate with the bacterial uptake in peritoneal macrophages, suggesting the different sensitivity of the experimental approaches with active uptake by flow cytometry being more sensitive. We supported the data on active bacterial uptake in peritoneal macrophages by flow cytometry with significant inhibition of phagocytosis in the presence of actin formation inhibitor, CCD (1); significantly decreased uptake at 4°C compared to the uptake at 32°C (2), and finally, with the evidence of the internalized bacterial strains by light microscopy. However, in order to assess the bactericidal properties in professional phagocytes, we performed bacterial killing assay. Absence of bacterial colonies in peritoneal macrophages exposed to the sera from fish challenged with *E. ictaluri* LAVs indicated that peritoneal macrophages efficiently killed WT *E. ictaluri* strain after 24 h of *in vitro* infection. Taken together, both approaches can provide valuable data on phagocytic properties and effective killing properties in professional phagocytes.

## Conclusion

Efficacious *E. ictaluri* LAVs are expected to induce active antigen uptake by phagocytosis and antigen presentation in catfish APCs such that infected monocytes/macrophages can initiate early activation of the innate immune system and acquired immunity mediated by T and B cells. Our study demonstrated that both vaccine candidates were endocytosed efficiently by catfish peritoneal macrophages showing significant increases in the intensity of uptake compared to their WT *E. ictaluri* counterpart. Importantly, both LAVs induced humoral immunity in the challenged fish resulting in significant increases in WT *E. ictaluri* uptake and bacterial destruction in catfish peritoneal macrophages *in vitro.* Our data on enhanced phagocytic capacity and effective killing ability of macrophages against ESC vaccine strains suggest the LAVs advantage to be processed and presented in the form of peptides to the specific lymphocytes of the adaptive immune system and support the importance of macrophage-mediated immunity against ESC in catfish.

## Author Contributions

LP and AK conceived and designed the experiments. LP and AK provided original idea of the study. AOK, HAb, HAh, and JP performed the experiments. LP and AK contributed reagents/materials/tools. AOK wrote the first draft of the manuscript and was involved in all aspects of the study. All authors were involved in critical interpretation of the data, manuscript revision, and final version approval.

## Conflict of Interest Statement

The authors declare that the research was conducted in the absence of any commercial or financial relationships that could be construed as a potential conflict of interest.

## Abbreviations

APCs: antigen-presenting cells
BHI: brain heart infusion
CCD: cytochalasin D
CCMM: Channel Catfish Macrophage Medium
CR: complement receptor
*E. ictaluri*: *Edwardsiella ictaluri*
ESC: enteric septicemia of catfish
HI: heat-inactivated
IN: intact serum
IP: intraperitoneal
LAV: live attenuated vaccine
MFI: mean fluorescence intensity
SPF: specific pathogen free
WT: wild-type.
